# Shoulder Pain and Cycle to Cycle Kinematic Spatial Variability during Recovery Phase in Manual Wheelchair Users: A Pilot Investigation

**DOI:** 10.1371/journal.pone.0089794

**Published:** 2014-03-10

**Authors:** Chandrasekaran Jayaraman, Yaejin Moon, Ian M. Rice, Elizabeth T. Hsiao Wecksler, Carolyn L. Beck, Jacob J. Sosnoff

**Affiliations:** 1 Department of Industrial and Enterprise Systems Engineering, University of Illinois at Urbana-Champaign, Urbana, Illinois, United States of America; 2 Department of Kinesiology and Community Health, University of Illinois at Urbana-Champaign, Urbana, Illinois, United States of America; 3 Department of Mechanical Science and Engineering, University of Illinois at Urbana-Champaign, Urbana, Illinois, United States of America; Delft University of Technology (TUDelft), Netherlands

## Abstract

Wheelchair propulsion plays a significant role in the development of shoulder pain in manual wheelchair users (MWU). However wheelchair propulsion metrics related to shoulder pain are not clearly understood. This investigation examined intra-individual kinematic spatial variability during semi-circular wheelchair propulsion as a function of shoulder pain in MWU. Data from 10 experienced adult MWU with spinal cord injury (5 with shoulder pain; 5 without shoulder pain) were analyzed in this study. Participants propelled their own wheelchairs on a dynamometer at 3 distinct speeds (self-selected, 0.7 m/s, 1.1 m/s) for 3 minutes at each speed. Motion capture data of the upper limbs were recorded. Intra-individual kinematic spatial variability of the steady state wrist motion during the recovery phase was determined using principal component analysis (PCA). The kinematic spatial variability was calculated at every 10% intervals (i.e at 11 interval points, from 0% to 100%) along the wrist recovery path.

**Results:**

Overall, spatial variability was found to be highest at the start and end of the recovery phase and lowest during the middle of the recovery path. Individuals with shoulder pain displayed significantly higher kinematic spatial variability than individuals without shoulder pain at the start (at 10% interval) of the recovery phase (*p*<.004).

**Conclusions:**

Analysis of intra-individual kinematic spatial variability during the recovery phase of manual wheelchair propulsion distinguished between those with and without shoulder pain. Variability analysis of wheelchair propulsion may offer a new approach to monitor the development and rehabilitation of shoulder pain.

## Introduction

It is estimated that over 2.8 million Americans use wheelchairs for mobility [1] with a majority (∼2 million) using manual wheelchairs [2]. Although wheelchair use has numerous benefits [3], the repetitive cyclic arm movement required for manual propulsion places a significant demand on the upper extremity, specifically the shoulder [4]–[7]. This increased demand often results in shoulder pain. Indeed up to 70% of manual wheelchair users report shoulder pain [5].

Shoulder pain in wheelchair users have been linked to difficulty performing activities of daily living, decreased physical activity and decreased quality of life [8]. Subsequently, it is imperative to understand the mechanisms that contribute to shoulder pain in manual wheelchair users so that appropriate interventions can be developed to prevent or minimize the effect of shoulder pain on function and thus reduce the risk of long-term upper extremity disability.

Investigations of mechanisms contributing to shoulder pain in wheelchair users have examined kinematic variables (i.e. arm motion parameters) of manual wheelchair propulsion [9]–[16]. While the research on shoulder pain and wheelchair propulsion has provided important information and has led to the development of clinical guidelines [9], [10], [17], [18], it has several potential limitations. First, there have been limited examinations of arm motion as a function of shoulder pain between individuals that utilize *the same propulsion pattern*. Second, research has mainly focused on the complete propulsion cycle and the push phase, but much less so on the recovery phase. Third, there have been minimal examinations of motor variability in wheelchair propulsion.

Before we elaborate further on each of these potential limitations, it is important to define a manual wheelchair propulsion cycle. A typical manual wheelchair propulsion cycle consists of a push phase (i.e. when the hand is in contact with the handrim/wheel) and a recovery phase (when the hand is off the handrim/wheel). During the push phase the arms are constrained to follow the handrim while during recovery phase the arms can adopt a variety of different movement patterns. Four typical propulsion pattern types have been observed based on the hand trajectory during the recovery phase of manual wheelchair propulsion [12], [13]. They are a semi-circular (SC) pattern, double loop pattern (DLOP), single loop pattern (SLOP) and an arc pattern [12], [13]. It has been suggested that using a SC pattern offers certain biomechanical advantages compared to the other pattern and hence could reduce the risk of shoulder injury in MWU [10], [12]–[16]. Although there are numerous investigations [10], [12]–[16] examining the advantages and disadvantage of different propulsion patterns, there are limited comparisons on kinematics of recovery phases between individuals who utilize the same propulsion pattern with and without shoulder pain.

Given that the recovery phase is the portion of the propulsion cycle that the arm is not constrained, it is logical to explore, if differences in the recovery kinematics exist in individuals with and without shoulder pain. Potentially these differences are important, because the trajectory through which the hands are brought back to start the subsequent push phase may reflect adaptive strategies used to handle the presence/effect of pain [19].

Moreover, it is believed that kinematic variability will be greatest during the recovery phase [20], [21], especially wrist movement variability (the distal joint of the arm segment) [22]–[24]. Although, not traditionally a marker of wheelchair propulsion there is growing evidence that variability is related to shoulder pain in MWU [25], [26]. Variability is an inherent characteristic of human movement [27]. Movement variability refers to the normal variations that occur within and across performance of motor tasks (e.g. wheelchair propulsion) [28]. Variability can occur both temporally and spatially and is an experimentally observable metric worthy of scientific inquiry, providing important information concerning the health of the neuromuscular system [22], [28]–[29]. Additionally, ergonomic research has revealed that motor variability in cyclic repetitive tasks is related to musculoskeletal pain both in the controlled laboratory environment and real world setting [19]. However, there is minimal information concerning kinematic variability of wheelchair propulsion and shoulder pain.

The purpose of this investigation was to determine if there were significant differences in kinematic spatial variability during the recovery phase of wheelchair propulsion between individuals with and without shoulder pain. Specifically, the kinematic spatial variability of the wrist motion during manual wheelchair recovery phase was examined. There are numerous techniques to quantify motor variability [30]. One approach is principal component analysis (PCA). PCA belongs to the factor analysis family and is a statistical decomposition technique used to identify patterns in data, thus highlighting data similarities and differences [31]. Although common in motor control/biomechanics research [32], [33], PCA has not been extensively applied to wheelchair biomechanics research.

Consistent with previous research on variability [19], it was hypothesized that manual wheelchair users with shoulder pain will have greater wrist kinematic spatial variability during the recovery phase when compared to manual wheelchair users without shoulder pain.

## Methods

### 2.1 Participants

Wheelchair propulsion data from ten individuals with spinal cord injury (four male, six female) from the Urbana-Champaign community was analyzed in this study. All the participants used a manual wheelchair as their primary means of ambulation for more than one year. Participants were classified into “with shoulder pain” (n = 5) and “without shoulder pain” (n = 5) groups based on their self-report (“Yes”/“No”- written response) of shoulder pain to our demographic questionnaire provided at the time of data collection.

### 2.2 Protocol

All experimental protocols in this study were approved by the University of Illinois at Urbana-Champaign institutional review board. Upon arrival to the laboratory, the experimental procedures were described to the participants and any questions they had regarding the protocol were clarified. Once participants understood the experimental procedures, they voluntarily signed the institutionally approved informed consent form. The participants then provided demographic information (age, height, weight, duration of wheelchair use, diagnosis, pain status, etc) and self-reported current status of shoulder pain (“Yes”/“No”). In addition to self reporting their current status of shoulder pain (“Yes”/“No”), participants also rated their current level of shoulder pain on a 10 cm visual analog scale (VAS) [34]. A score of 0 cm indicated that the participant was not experiencing any shoulder pain at the time of data collection and a score of 10 cm indicated existence of high level of shoulder pain at the time of data collection.

Following the collection of all volunteer demographic and shoulder pain data, the participants' personal wheelchair was fitted bilaterally with 25 inch diameter SMARTWheels (Three Rivers Holdings LLC; AZ, USA). Individuals' upper extremity kinematics is not significantly affected by attaching/testing with different SMARTWheel sizes [35]. Attaching the SMARTWheels to the participant's personal wheelchair does not change the wheel placement alignment or camber [36]. The participant's wheelchair was then secured to a single drum dynamometer with a fly wheel and tie-down system [37].

### 2.3 Kinematic data collection: motion capture

Based on the International Society of Biomechanics (ISB) recommendations [38], 18 reflective markers were attached at specific bony landmarks to define the trunk, upper arm, forearm, hand, sternum and the jaw: these included sternal notch, C7 vertebrae, T3 vertebrae, T6 vertebrae and bilaterally at the mandible, third metacarpophalangeal joint, radial styloid ulnar styloid, olecronon, lateral epicondyl and the acromion process. Two reflective markers, one on the wheel center and other on the wheel spoke were placed on each of the wheels. Kinematic data were collected using a 10 camera motion capture system (Cortex 2.5, Motion Analysis Co.; Santa Rosa, CA, USA) at a sampling rate of 100 Hz.

Participants were asked to propel at constant speeds for three separate 3 minute trials at 1.1 m/s (fast), 0.7 m/s (slow) and self-select (∼0.88 m/s) speeds. The sequence of speeds was randomized for each participant. A speedometer was used to provide real-time visual feedback to the subjects while kinetic data were collected bilaterally at 100 Hz. Sufficient rest and recovery was provided between each trial. Subjects were given time to acclimate to the dynamometer and propulsion speed before the beginning of each trial. A force plate was used to measure the weight of participants (AMTI, Inc., Watertown, MA, USA).

### 2.4 Kinematic data post processing

Kinematic and kinetic data were collected for each trial. The motion data were post processed and any missing intermediate marker data points were fit using a cubic interpolation. Interpolation was accomplished by using the post processing module in the Cortex 2.5 Motion Analysis software. The kinetic data from SMARTwheel were used to identify the propulsion and recovery phases of each stroke. This study focused on the recovery behavior; therefore, the start and end points of the recovery phase were located based on when the moment applied to the hand rim (Mz) was lower or greater than 1 Nm, respectively, for at least 10 ms [39], [40]. For the shoulder pain group, the kinematic data belonging to the side with highest shoulder pain level was analyzed (right (n = 4) and left (n = 1)), while the kinematic data of the dominant hand (right (n = 4) and left (n = 1)) was analyzed for the group without shoulder pain.

The post processed motion data were filtered using a fourth-order low-pass Butterworth filter with 7 Hz cut-off frequency to remove the high frequency components [41]. The hand's (third metacarpophalangeal joint) sagittal plane displacement was used to classify the propulsion pattern type (semi-circular). While wrist motion data during the recovery phase were used to compute the kinematic spatial variability. The wrist motion data was computed as the mid-point of the radial styloid (RS) and ulnar styloid (US) arm segment marker coordinates [42]. Cycles having any data points outside of 3 standard deviations were excluded from the analysis [43], [44]. The motion data were separated into propulsion and recovery phases. Data samples belonging to the first five cycles and the last cycle were removed from each trial [45], [46]. The wrist position (sagittal plane) for each recovery phase were time normalized to 100 data points using a shape preserving cubic spline interpolation. The range of motion (ROM) was computed by quantifying the arc length travelled by the wrist during the recovery phase. The cycle-to-cycle mean power output at the hand-rim was computed from the SMARTWheel data [39]. We used PCA to observe the underlying spatial variability (i.e., as determined by the variance structure in the data) of the wrist recovery trajectory during wheelchair propulsion in individuals with and without shoulder pain. For details on PCA computation please refer to the Appendix S1.

**Figure 1:**
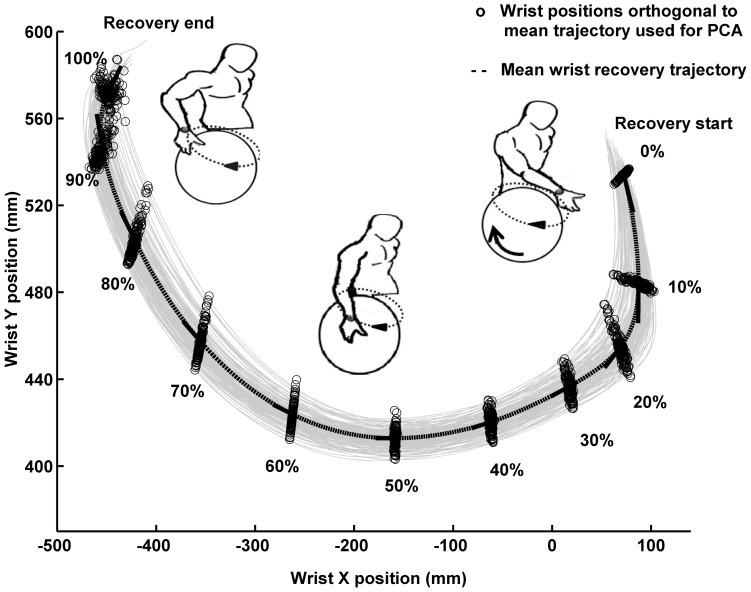
Sample steady-state wrist recovery trajectories for a semi-circular propulsion pattern. Wrist cycle-to-cycle recovery trajectories (“grey solid lines”) for a participant with shoulder pain during a three minute trial at fast speed (1.11(.05) m/s). The mean wrist recovery trajectory is shown by the bold dashed line at center (“**- -**”). The wrist positions orthogonal to mean recovery trajectory for which PCA was computed (0% to 100% at every 10% interval along the recovery path) is denoted by (“o”).

The mean wrist recovery trajectory was computed for each trial from the normalized data. The recovery wrist positions orthogonal to the mean path at each 10% spatial interval for every recovery phase was calculated from the normalized data (Figure 1). These wrist positions orthogonal to mean path were determined such that the cross product between the mean wrist recovery trajectory at a chosen spatial interval and the line connecting that wrist position to the mean trajectory should be near zero. Principal components were computed for the recovery wrist position in directions orthogonal to mean recovery path at equally spaced (10%) intervals (from start of recovery 0% to end of recovery 100%) (Figure 1) [43], [44]. Since we computed the principal components orthogonal to the mean trajectory, the problem was essentially reduced to two dimensions leaving us with two components, first principal component (PC_1_) and second principal component (PC_2_) each denoting the magnitude of variance in the directions orthogonal to the mean path. The square root of PC_1_ at each 10% interval along the recovery path was used as a metric of kinematic spatial variability [43], [44]. For brevity, we refer to the square root of PC_1_ as kinematic spatial variability.

### 2.5 Statistical analyses

All statistical data analyses were conducted using SPSS (version 21, IBM, Inc.). Series of two tailed independent t-test was conducted to verify if there were statistically significant between group differences in demographic information (age, wheelchair experience, weight and shoulder pain scores) and mean power output at hand-rim. Differences in gender composition between groups were evaluated using a X^2^ test.

A 2 (group)×3 (speed)×11 (interval points) repeated measure ANOVA with shoulder pain group as the between group factor and speed conditions and interval along recovery trajectory (0% to 100%) as the within subject factors was used to analyzed kinematic spatial variability. Significance level was set at 0.05. When appropriate, a Bonferroni correction was made. To further examine the interaction between pain group and interval, 11 separate univariate ANOVA's with pain group as the between subject factor were conducted on kinematic spatial variability within each interval. A corrected significance level was set at 0.004 for the univariate test and a Bonferroni correction was made when appropriate. All values are reported as Mean(SD) unless otherwise noted.

## Results

### 3.1 Demographics

Demographic statistics are furnished in Table 1. There were no statistically significant group differences in gender composition, age, torso height, wheelchair experience and body weight, (*p*'s>0.05). 80% of the participants (4 out of 5 in each group) had spinal injury at and below level T1. Per design, self-reported shoulder pain scores were significantly greater, [t (8) = 2.56, *p*<0.05] in the shoulder pain group (3.1(2.7)) compared to the no-pain group (0(0)).

**Table 1:** Participant demographics.

Characteristics	Pain (n = 5)	No pain (n = 5)
	Mean(SD)	Mean(SD)
Gender (M/F)	2/3	2/3
Spinal injury demographics	Birth defect T11-L2(n = 1), T8 paraplegic(n = 1), Spinal cyst - T6(n = 1), Sacral agenesis(n = 1), Spina bifidia(n = 1)	Transverse myelitis T9/L2 (n = 1), T9 paraplegic(n = 1), Spinal AVM(T6-T9)(n = 1), C7(n = 1), Birth defect T6(n = 1)
Age(years)	28.8(15.06)	24.8(7.2)
Body weight(lb)	159.7(65.2)	122.7(43.2)
Experience using wheelchair(years)	20.8(4.9)	14(5.5)
Torso height (mm)	375(57.66)	406(75.03)
VAS (self-reported current pain score)*	3.1(2.7)*	0.0(0.0)*

Note:

*p<0.05.

### 3.2 Mean power output at handrim

Statistical analysis revealed that there were no significant group differences in mean power output across the three speed conditions tested (*p's*>0.05). The mean power output as a function of speed is reported in Table 2.

**Table 2:** Power output as a function of group and speed.

Speed condition	Power(W)	
	Pain group	No pain group
Fast	17.31(6.23)	12.89(6.45)
Self selected	15.34(6.61)	11.94(5.58)
Slow	14.04(5.36)	11.49(5.39)

Note: Values are Mean (SD).

### 3.3 Mean ROM measure

Statistical analysis revealed that there was a main effect of speed on range of motion (ROM) [F (1, 8) = 8.0, *p* = 0.02, η^2^ = 0.5]. Higher mean ROM was observed with increasing trial speed (fast (1.11(.05)m/s)∶703.5(85.08) mm; self (0.88(.13)m/s)∶681.7(78.82) mm; slow (0.72(.05)m/s)∶678.1(74.64) mm). No significant differences in mean ROM between groups were observed as a function of shoulder pain [F (1, 8) = 1.4, *p* = 0.30, η^2^ = 0.1] (Pain group : 658.8 (59.8) mm, No Pain group : 716.7(92.1) mm).

### 3.4 Recovery stroke kinematic spatial variability

Figure 1 illustrates a sample recovery trajectory of a representative participant with shoulder pain and the 10% intervals along the wrist recovery path where the PCA's were computed. For all participants, irrespective of pain status, PC_1_ accounted for more than 80% of the variance while PC_2_ accounted for the rest. Based on this observation, only PC_1_ was used for calculating kinematic spatial variability. References for this criterion is provided in the supplementary material where we discuss about the PCA computation.

**Figure 2:**
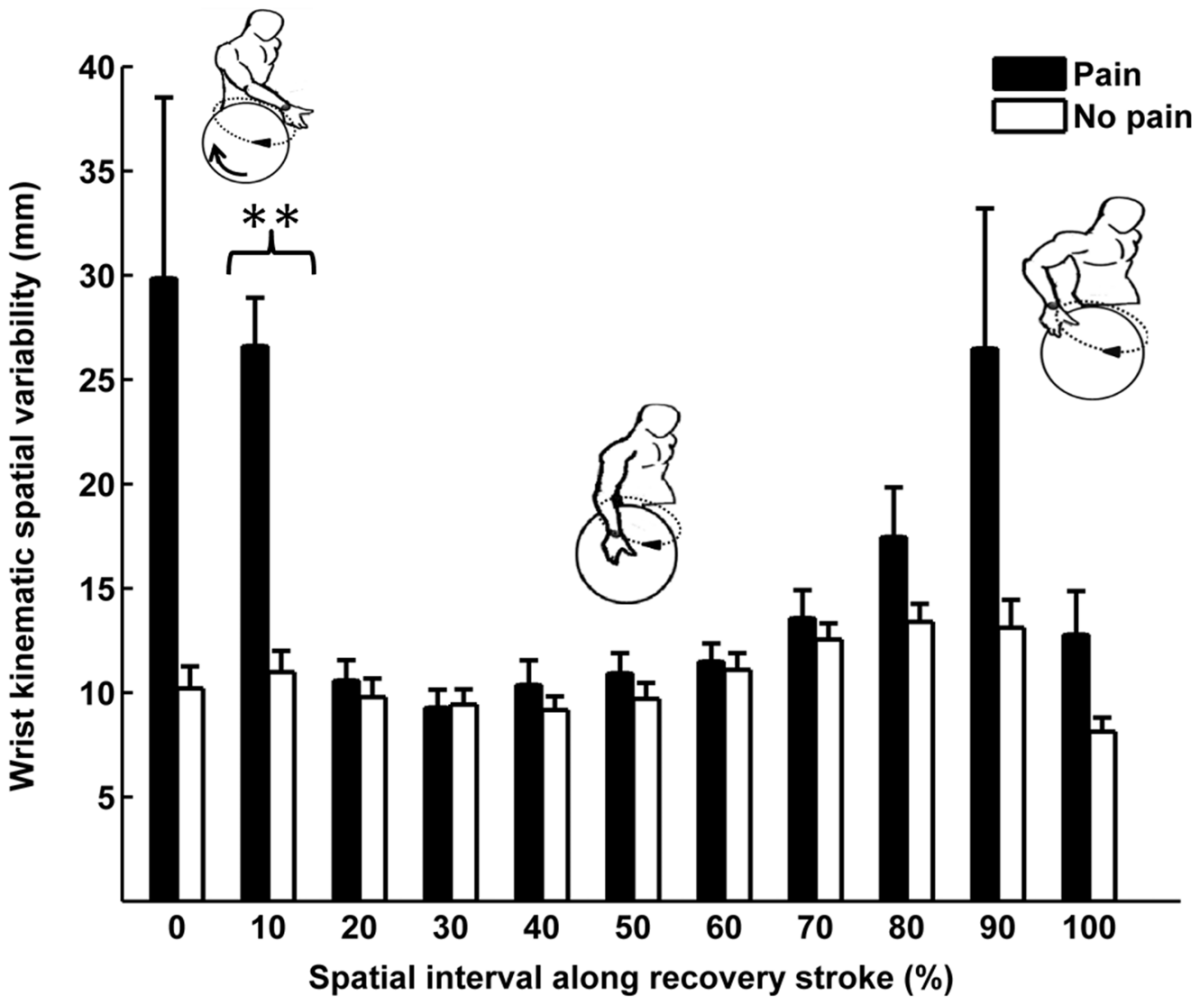
Recovery phase wrist spatial kinematic variability at steady state propulsion between shoulder pain groups for a semi-circular pattern (Mean(SE)). Wrist kinematic spatial variability at every 10% interval along the recovery phase as a function of shoulder pain group. Mean(SE) values collapsed across speed conditions. **significant difference (*p*<.004). SE – Standard error.

Figure 2 illustrates the recovery phase wrist kinematic spatial variability for groups with and without shoulder pain, collapsed across speed conditions. On average for both the groups, the kinematic spatial variability approximated an asymmetric U-shaped curve having greater values at the start and end regions of the recovery phase, with smaller values occurring in-between 30% and 50% along the recovery path.

There was no main effect of speed on kinematic variability [F (2, 16) = 0.15, *p*>0.05, η^2^ = 0.02]. Further, there was no significant interaction between pain group and speed [F (2, 16) = 0.17, *p*>0.05, η^2^ = 0.02]. There was a significant main effect of interval on kinematic variability [F (1, 80) = 2.84, *p*<0.05, η^2^ = 0.26]. There was also a significant interaction between pain group and interval F (1, 80) = 1.96, *p*<0.05, η^2^ = 0.20]. In order to examine the pain group by interval interactions, univariate ANOVA's with pain group as the between subject factor were conducted on each of the 11 intervals points. Between-group intra individual kinematic spatial variability was significantly different at the 10% interval (Figure 2), [F (1, 28) _10%_ = 33.51, *p<0.000*, η^2^ = 0.55, observed power = 1.00] with the shoulder pain group having greater kinematic spatial variability than the group without shoulder pain. There were no significant group differences in kinematic spatial variability (*p*'s>0.004) at wrist locations other than in the 10% interval.

## Discussion

This pilot investigation was designed to begin to address three gaps in the wheelchair propulsion literature. Namely, 1) The lack of information concerning kinematics of individuals with and without shoulder pain who utilize the same propulsion pattern; 2) minimal investigation of the recovery phase of propulsion; and 3) lack of information concerning variability in wheelchair propulsion. Specifically, it examined the relation between shoulder pain and kinematic spatial variability of steady state wrist motion during the recovery phase in manual wheelchair propulsion of individuals who utilized a semicircular pattern. Overall it was found that participants with shoulder pain had significantly greater kinematic spatial variability at the beginning of the recovery phase than those without shoulder pain. This observation is consistent with the evidence that motor variability is related to musculoskeletal pain [19], [47]. The observations highlight that the including examination of recovery phase kinematic variability parameters may provide further insight into the shoulder pain in manual wheelchair users.

Previous wheelchair biomechanics researches have mainly focused on mean propulsion parameters [48], [49]. Consistent with their observations [48], [49], the current results showed no significant difference in mean wrist ROM between shoulder pain groups. In contrast, the examination of kinematic spatial variability was able to identify significant differences between pain groups. This observation demonstrates that variability is a more sensitive identifier of shoulder pain in MWU.

In contrast to significant group effect observed as a function of shoulder pain, there was no effect of propulsion speeds on kinematic spatial variability. The lack of speed effect may stem from the participants using a larger ROM of shoulder joint and increased cadence to propel at faster speeds [20]. It is also possible that the speeds utilized here were not sufficiently distinct from each other to elicit a significant effect.

Although the association between variability and self-reported pain is relatively novel within wheelchair biomechanics research, it is consistent with motor control/biomechanics/ergonomics research that has demonstrated that variability can play a functional role in the prevention and/or development of injury [47]. For instance, ergonomic investigations have reported an increase in arm movement variability in individuals with musculoskeletal pain performing occupational tasks [19], [50]. Additionally, studies examining repetitive reaching tasks demonstrate that subjects with shoulder pain exhibited higher relative variability in their kinematics than those without pain [51], [52].

There are several potential reasons why persons with shoulder pain exhibited greater variability in their movement. One possible explanation could be that the group with shoulder pain utilized a pain minimizing strategy to reduce momentary shoulder pain during the beginning of the recovery phase [50]–[53]. Specifically, the group with shoulder pain could have adapted a compensatory strategy (more spatial variability in a specific direction) to minimize momentary pain effects while propelling their wheelchair. The higher kinematic spatial variability could also have resulted from contributions from alternate muscle groups to accomplish the task with less shoulder discomfort. Congruent with this possibility, studies on repetitive motion task in persons with neck/shoulder pain observed that the shoulder pain group's adaptability was primarily spatial in nature [53]. Muscle electrical activity was not recorded in the current study so it is merely a speculation that the contributions from muscles were different between the pain and no pain group.

An important observation that requires further clarification is why significant between groups differences only occurred at the 10% location of the recovery phase. This aspect of the recovery phase encompasses the transition dynamics from a closed chain to open chain movement. Specifically, in handrim wheelchair propulsion, during the push phase the arm segments form a closed chain as the hand is fixed to the (rotating) rim [54]. However, during recovery phase the arm is not constrained to follow a guided movement determined by the fixed handrim (e.g. open chain movement). Indeed, similar observations of higher kinematic variability during recovery kinematics near this transition point have been reported previously [20], [21].

Additionally, during the start of recovery phase, a movement goal is to counteract (absorb) the reactive force/moment resulting from the push phase. Specifically, the shoulder joint has to decelerate the forward movement of the arm joints, coordinate a directional change of arm segment movement to accelerate towards end of recovery phase. While during the end of recovery phase the goal is to decelerate the arm joints to prepare for the next push phase. The main function of the shoulder towards the end of the recovery phase has been reported to involve large amount of elbow stabilization [11]. For instance, [45], studied the propulsion and recovery power requirements of arm joints in experienced MWU with spinal injury and reported near zero power at shoulder joint during the end of the recovery phase. For a given propulsion task, the goal at the start of the recovery phase (∼10%), immediately after experiencing a push phase (i.e. shoulder joint relieved from applying force/moment) is particularly more complex in terms of timely coordination of multiple events (i.e arm joint deceleration and direction change while maintaining a certain rhythm of arm movement) in addition to the power absorption. This may be a reason why we observed significant between-group differences in wrist spatial variability only during the start of the recovery phase (10%). The group with shoulder pain may have used a spatial strategy (i.e greater kinematic spatial variability) to overcome the difficulty (i.e minimizing discomfort) while coordinating and controlling such complex task given their shoulder pain). Similar adaptive behavior have been observed in ergonomic studies that analyzed repetitive motion task in persons with neck/shoulder pain and found that the pain group's adaptability was primarily spatial in nature [53].

Our results and discussion so far, suggest that analyzing variability measure of recovery phase kinematics is relevant in the context of shoulder pain in MWU. While we acknowledge that analyzing variability measures during the push phase of wheelchair propulsion bears importance [25], [26], we recommend inclusion of variability measures of recovery phase variables as well. This provides complementary information to understand individual differences in variability which arise as an aftermath of the push phase dynamics [26]. As mentioned previously variability in movement will be greatest at the distal segments during open chain movements [20]–[24]. Indeed, examining variability of open chain movements has been an experimental centerpiece of motor control research [22].

Despite the novel observations of this investigation, there were some limitations. The small sample size raises issues with generalizability. Obviously, our findings should be replicated in larger samples. Despite the sample size limitation, we found significant differences in wrist kinematic spatial variability as a function of shoulder pain between the two groups. Our sample size was to small to investigate the influence of specific injury characteristics on the kinematic spatial variability. Future studies with larger sample size can focus on groups with same injury demographics.

The current data and experimental design cannot address whether increased variability in the recovery kinematics results from shoulder pain or vice versa. Only a longitudinal design could answer these questions clearly. More than 60% of the sample in the current study were college students and had congenital spinal cord injury, which may impact the pathogenesis of musculoskeletal pain [55]. Information on wrist pain demographics was not collected. But we think it is reasonable to expect that wrist pain is unlikely to influence wrist kinematics in the sagittal plane during the recovery phase since the wrist experiences minimal forces/moments. Not including a physical examination to assess the nature of shoulder pain (impingement or neuropathic) is another limitation.

All participants in the current investigation demonstrated a semi-circular propulsion pattern. Although, this is a methodological strength of the current investigation, it is not clear if other propulsion patterns would demonstrate that same variability profile and if there would be a difference in spatial variability between those with and without shoulder pain utilizing another propulsion pattern (i.e. double loop, arc and single loop [12], [13]). Additionally, none of the participants from this study used a 25 inch wheel in their personal wheelchair. It is not known if the kinematic spatial variability would be affected due to testing them on a 25 inch wheel. However, a recent investigation [35] observed no significant differences in upper extremity kinematics when using different SMARTWheel sizes. Yet, Mason and colleagues did not report variability metrics. The effect of wheelchair configuration on kinematic variability is a topic worthy of future investigation.

A last limitation involves the roller dynamometer setup utilized here. It was not equipped to measure power output at different speed conditions which could be a confounding factor. But given that the body weight and propulsion speed was not significantly different, it is reasonable to assume that power output differences had minimal influence on our results.

## Conclusions

Individuals with shoulder pain had higher kinematic spatial variability during the beginning of recovery stroke as compared to those without pain. To our knowledge, this is one of the first investigations to document an association between variability measure of recovery kinematics and symptomatic shoulder pain in manual wheelchair users. Integrating the recovery kinematic spatial variability into clinical and rehabilitation practice may pave the way for new interventions for tracking treating, and/or preventing shoulder related pathologies in the manual wheelchair population.

## Supporting Information

Appendix S1 (DOCX)
