# Supplementary material for: Shoulder Pain and Cycle to Cycle Kinematic Spatial Variability during Recovery Phase in Manual Wheelchair Users: A Pilot Investigation
Source: PLoS One. 2014 Mar 10;9(3):e89794. doi: 10.1371/journal.pone.0089794 (PMC3948626; doi:10.1371/journal.pone.0089794)
Supplement: Appendix S1 — (DOCX) [file pone.0089794.s001.docx]

**Appendix S1**

**Details of principal component analysis computation**

A PCA computation for a given data matrix X consists of three main steps: (1) computing the *relationship matrix* (i.e., the covariance matrix C) which captures the interrelationship between the variables. The expression for covariance matrix, C is given by

where Y is the mean-detrended data matrix, Y^T^ is the transposed mean-detrended data matrix and k is the row length of the data matrix X; (2) extracting and retaining the component factors that account for the greatest variance, based on an eigenvalue decomposition of C; (3) performing a varimax rotation [1] for allocating the variances between factors. The number of components resulting from applying PCA is always equal to or less than the number of input variables, with the first component being the factor which accounts for the greatest variance seen in the data, and the following components being those factors with remaining highest variance subject to being uncorrelated with previous components. Only those principal components (PCs) that have large magnitude are retained; the remaining components are discarded as random noise [2-4]. For a more detailed mathematical description of PCA and its applicability to kinematic data analysis in biomechanics, the reader is referred to [5].

**References**

1. Kaiser H (1958) The varimax criterion for analytic rotation in factor analysis. Psychometrika 23: 187-200
2. Cattell RB (1966) The Scree Test For The Number Of Factors. Multivariate Behavioral Research 1: 245-276.
3. Cattell RBJ, Joseph (1967) A general plasmode (no. 30-10-5-2) for factor analytic exercises and research. Multivariate Behavioral Research Monographs Vol 67-3.
4. [Todd CH](http://www.amazon.com/s/ref=ntt_athr_dp_sr_1?_encoding=UTF8&field-author=Todd%20C.%20Handy&search-alias=books&sort=relevancerank) (2004) Event related potential, A methods handbook. Chapter 9.
5. Daffertshofer A, Lamoth CJ, Meijer OG, Beek PJ (2004) PCA in studying coordination and variability: a tutorial. Clin Biomech (Bristol, Avon) 19: 415-428.
